# Phytosterol oxidation products (POP) in foods with added phytosterols and estimation of their daily intake: A literature review

**DOI:** 10.1002/ejlt.201500368

**Published:** 2016-01-21

**Authors:** Yuguang Lin, Diny Knol, Elke A. Trautwein

**Affiliations:** ^1^ Unilever Research and Development VlaardingenThe Netherlands

**Keywords:** Foods, Heating, Oxidised phytosterols, Plant stanols, Plant sterols

## Abstract

To evaluate the content of phytosterol oxidation products (POP) of foods with added phytosterols, in total 14 studies measuring POP contents of foods with added phytosterols were systematically reviewed. In non‐heated or stored foods, POP contents were low, ranging from (medians) 0.03–3.6 mg/100 g with corresponding oxidation rates of phytosterols (ORP) of 0.03–0.06%. In fat‐based foods with 8% of added free plant sterols (FPS), plant sterol esters (PSE) or plant stanol esters (PAE) pan‐fried at 160–200°C for 5–10 min, median POP contents were 72.0, 38.1, and 4.9 mg/100 g, respectively, with a median ORP of 0.90, 0.48, and 0.06%. Hence resistance to thermal oxidation was in the order of PAE > PSE > FPS. POP formation was highest in enriched butter followed by margarine and rapeseed oil. In margarines with 7.5–10.5% added PSE oven‐heated at 140–200°C for 5–30 min, median POP content was 0.3 mg/100 g. Further heating under same temperature conditions but for 60–120 min markedly increased POP formation to 384.3 mg/100 g. Estimated daily upper POP intake was 47.7 mg/d (equivalent to 0.69 mg/kg BW/d) for foods with added PSE and 78.3 mg/d (equivalent to 1.12 mg/kg BW/d) for foods with added FPS as calculated by multiplying the advised upper daily phytosterol intake of 3 g/d with the 90% quantile values of ORP. In conclusion, heating temperature and time, chemical form of phytosterols added and the food matrix are determinants of POP formation in foods with added phytosterols, leading to an increase in POP contents.

**Practical applications:** Phytosterol oxidation products (POP) are formed in foods containing phytosterols especially when exposed to heat treatment. This review summarising POP contents in foods with added phytosterols in their free and esterified forms reveals that heating temperature and time, the chemical form of phytosterols added and the food matrix itself are determinants of POP formation with heating temperature and time having the biggest impact. The estimated upper daily intakes of POP is 78.3 mg/d for fat‐based products with added free plant sterols and 47.7 mg/d for fat‐based products with added plant sterol esters.

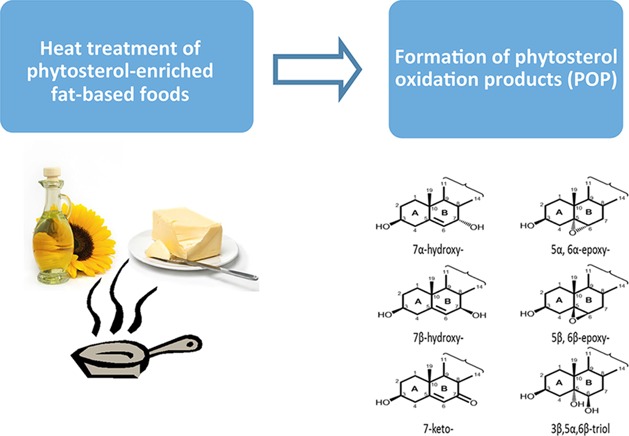

Phytosterols in foods are susceptible to oxidation to form phytosterol oxidation products (POP). This review summarizes literature data regarding POP contents of foods with added phytosterols that were exposed to storage and heat treatments.

**Abbreviations**
COP: cholesterol oxidation products
EFSA: European Food Safety Authority
FPS: free plant sterols
ORP: oxidation rates of phytosterols
PAE: plant stanol esters
POP: phytosterols oxidation products
PSE: plant sterol esters

## 1 Introduction

**Figure 1:**
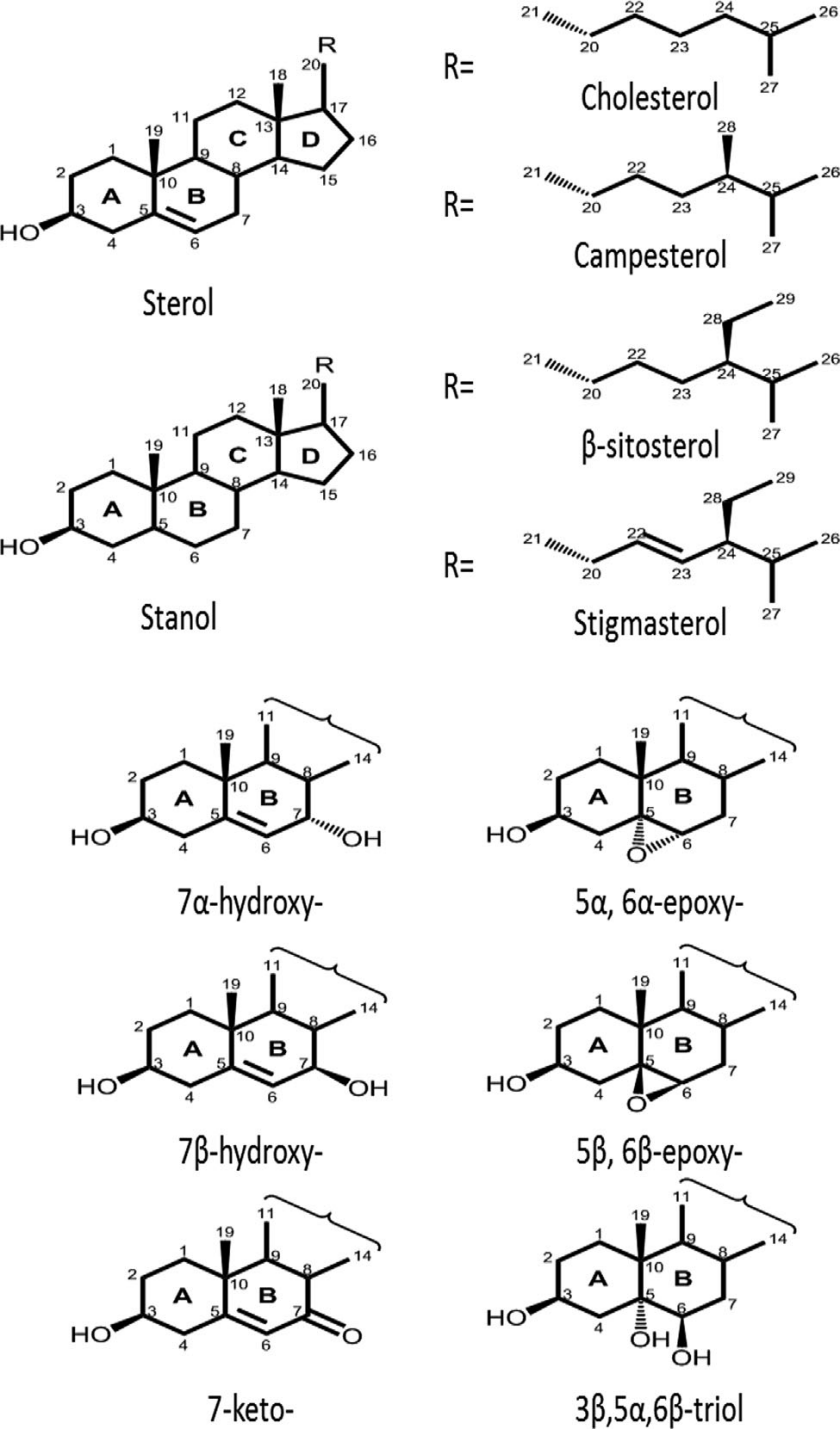
Chemical structures of sterols and their oxidation products.

Plant sterols and stanols (collectively referred to as phytosterols) are naturally occurring compounds found in all plant‐derived foods, especially being rich in vegetable oils and products made from them. Plant sterols and stanols differ in their chemical structures as stanols lack a double bond in the steroid ring structure (Fig. 1). Plant sterols and stanols account for 96% and 4%, respectively, of the naturally occurring phytosterols in the human diet 1. With habitual diets, the daily intake of naturally occurring phytosterols ranges between 178 and 463 mg/day 1. Due to the well‐established cholesterol lowering effect of phytosterols 2, commercial food products, for example, margarine and dairy products, with added phytosterols either in their free or esterified form, have been marketed for decades.

Sterols, including cholesterol 3, and plant sterols 4 and to a lesser extent plant stanols 5, are susceptible to oxidation to form the corresponding cholesterol oxidation products (COP) and phytosterol oxidation products (POP). It is well known that cholesterol oxidation can take place via both enzymatic oxidation and autoxidation (non‐enzymatic oxidation), generating oxidation at the side‐chain and steroid ring of the cholesterol molecule 3. Phytosterols differ from cholesterol at their side‐chain with an additional ethyl or methyl group at C24 and/or a double bound at the C22 position (Fig. 1). Thus, phytosterols are poor substrates for cellular enzymes responsible for the enzymatic oxidation of cholesterol 3. Therefore, enzymatic oxidation may not play an important role in catalysing phytosterol oxidation in the body 3. Clearly, autoxidation of phytosterols might be the major source of POP formation. Autoxidation of phytosterols can take place not only in the body but also in foods during food storage and especially during heating processes such as cooking, frying and baking, leading to the formation of POP in foods. The autoxidation of sterols (cholesterol and plant sterols) generates steroid ring oxidation products, commonly 7‐hydroxy‐(7‐OH)‐, 7‐keto‐, 5,6‐epoxy‐ and 3,5,6‐triol‐derivatives of sterols (Fig. 1). Since plant stanols have no double bond at the C5,6 position, plant stanols are not converted into 5,6‐epoxy‐ and triol‐derivatives 5.

An increasing amount of research shows that POP have biological effects different from their corresponding parent phytosterols, which have been summarised in several literature reviews 3, 6, 7, 8. Briefly, the reported biological effects of POP include modulating lipid and glucose metabolism, cell apoptosis, inflammation processes and immune reactions, which may be linked to either beneficial effects (e.g., anti‐diabetic and anti‐tumour) or detrimental effects (e.g., atherogenicity, cytotoxicity, pro‐inflammation, mitochondrial dysfunction, glutathione depletion, and oxidative stress) 3, 6, 7, 8, 9. A main health concern related to a harmful effect of POP raised in the literature is the speculation that POP, like COP, may be more atherogenic than cholesterol 4, 10, 11 because of their molecular structure similarity. COP were shown to contribute to the pathological processes of atherosclerosis 10, 11. In vitro and animal studies as reviewed by O'Callaghan et al. 4 and Otaegui‐Arrazola et al. 7 have shown similar atherogenic activities of COP and POP. For example, a recent study in female LDL‐receptor deficient mice showed that both COP‐ and POP‐containing diets enhanced atherosclerosis compared to the control diet 12. However, other study data do not support the similar atherogenic activities of POP and COP 13, 14. Clear evidence for atherogenic effect of POP in humans is lacking.

Fasting plasma POP concentrations in healthy subjects are in the range of 4.18–109 μg/L (0.01–0.26 μM/L) 9. Baumgartner et al. reported that daily intake of 3 g phytosterols (in the form of plant sterol esters (PSE) for 4 wk did not increase fasting plasma POP concentration 15, while PSE intake increased plasma 7β‐hydroxy‐phytosterols postprandially by 0.1 μg/L compared to the control diet 16. Husche et al. 17 found that serum 7β‐hydroxy‐sitosterol increased by about 1.15 μg/L after intake of 3 g/d phytosterols from a PSE‐enriched margarine. However, these data do not allow to draw a clear conclusion about the contribution of dietary POP to plasma POP concentrations since data on absorption rates of POP in humans are not yet available. Studies in rats show that absorption rates ranged between 2.7% and 15.9% 14, 18, suggesting that dietary POP might be a determinant of plasma POP concentrations next to endogenous formation. Due to the possible (beneficial and detrimental) implications of POP on human health and the natural occurrence of autoxidation of phytosterols in foods, it is of interest to systematically assess POP contents in foods, especially in those enriched with phytosterols. Furthermore, most of the reported studies regarding physiological or pathological effects of POP are based on in vitro and animal studies using high concentrations of POP. For example, in studies with animals 25–74 mg/kg BW/d of POP were administered 12, 14, 19, 20. It has not been reviewed whether such amounts are realistically ingested by humans from consumption of foods with added phytosterols that are prepared under typical heating conditions like cooking and baking. POP contents in foods with or without added phytosterols have recently been reviewed 9. The aims of this review are to i) systematically summarize available data on POP contents in foods with added phytosterols in either their free or esterified form, ii) to assess factors that affect POP formation and their contents in foods under various storage and heat treatment conditions, and iii) to estimate daily POP intakes when consuming foods with added phytosterols.

## 2 Methods

### 2.1 Literature search strategy

To retrieve relevant publications, three literature databases (Food Science & Technology Abstracts, Ovid MEDLINE(R) In‐Process & Other Non‐Indexed Citations, CAB Abstracts) were systematically searched through Feb 2015 by using the Wolters Kluwer OvidSP search tool. The search strings (terms) used were (POP or oxi* or oxy*) AND (phytosterol* or phyto sterol* or plantsterol* or plant sterol* or sitosterol* or campesterol* or stigmasterol* or brassicasterol*) AND (food* or margarine* or vegetable oil* or dairy product* or chocolate or baking product* or vegetable*).

### 2.2 Selection of eligible studies

With the systematic search, 776 papers in total were identified. Selection of eligible papers was done in two rounds. In the first round, titles and abstracts were screened and those studies not fulfilling the predefined inclusion criteria were excluded. These inclusion criteria were 1) studies conducted with foods and 2) POP contents in these foods measured. After removing duplications and excluding those publications that did not meet the criteria, the number of potential eligible papers was reduced from 776 to 87.

In the second selection round, full publications were read to judge their eligibility. Seventy‐four papers were then excluded based on the following predefined exclusion criteria: 1) Studies with non‐ phytosterol‐added foods. This is because we aimed to assess POP contents in foods with added phytosterols. 2) To avoid underestimation of the total POP content, studies were excluded that measured or described less than two groups of individual POP of the four main POP groups. These are (i) 7α‐ and 7β‐hydroxy‐phytosterols, (ii) 7‐keto‐phytosterols, (iii) 5α,6α‐ and 5β,6β‐epoxy‐phytosterols, and (iv) 3β,5α,6β‐triol‐phytosterols. 3) From the reported data, the POP contents per 100 g edible portion of foods could be calculated, which allows the comparison of different studies. For our assessment, POP content per 100 g food was used as the parameter for summarizing data and comparing data amongst reported studies. 4) Review papers or book chapters were excluded to avoid double data collection.

**Figure 2:**
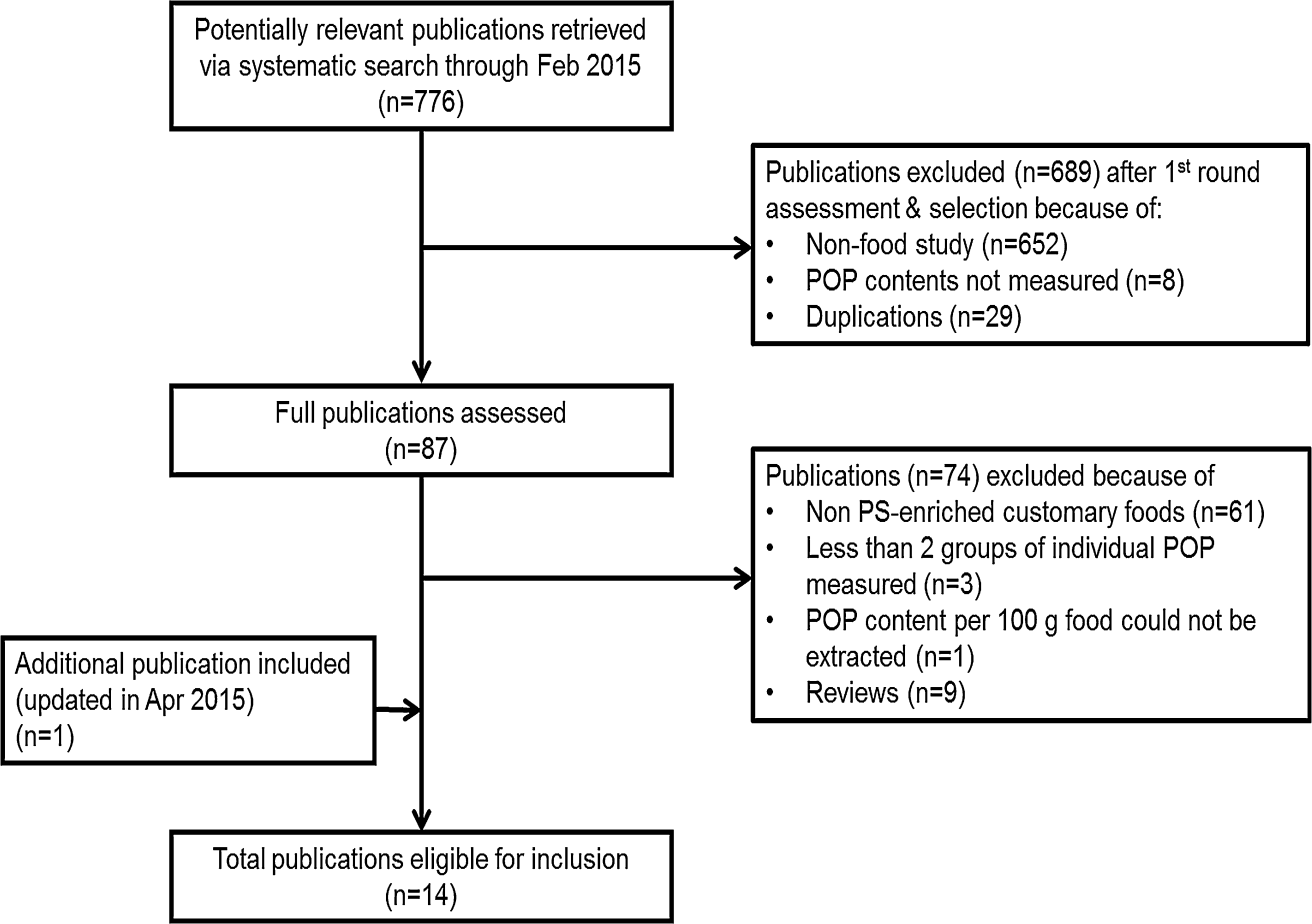
Flow chart of identification and selection process for eligible publications.

During preparation of the manuscript, one recent publication from April 2015, was defined to be eligible for this review. In the end, a total of 14 eligible publications/studies were included 15, 17, 21, 22, 23, 24, 25, 26, 27, 28, 29, 30, 31, 32. The selection process of eligible papers is summarised in a flow chart (Fig. 2).

### 2.3 Data extraction and transformation

We collected the following data from the eligible studies: 1) study identification (author, publication year); 2) food characteristics (format, source, phytosterols contents); 3) treatment characteristics (storage, exposure to air or sunlight, temperature, heating time, and method, for example, oven‐ or pan‐heating); and chemical and analytical methods for POP measurement. These data were collected in light of assessing factors that may affect POP formation and hence their contents in foods. We have not assessed the quality of the chemical analysis methods of the individual studies, considering that making such a judgement of quality is rather subjective by taking into account the lack of an agreed harmonised method for analysis of POPs in foods.

Data of both phytosterol and POP contents in foods were extracted from the eligible publications. Phytosterols refer to the sum of the reported individual plant sterols and plant stanols, respectively. Plant stanol products used in the reported studies contained small amounts (1–10%) of plant sterols. POP refer to the sum of all the POP derived from the individual phytosterols. Typical POP in foods are 7α/β‐hydroxy‐ (7‐OH‐), 7‐keto‐, 5,6α/β‐epoxy‐ (epoxy‐), and 3,5,6‐triol‐phytosterols (triols) 24, 33. For plant stanols, POP was based on the sum of the formation of 7‐OH‐, 6α‐hydroxy‐ and 15α‐hydroxy‐phytostanols 24, 34.

For phytosterol contents in foods, only data at baseline, that is, before treatment were extracted because the majority of the reported studies did not measure phytosterol concentrations after thermal or storage treatments. The oxidation rate of phytosterols (ORP) was calculated according to: ORP in% = content of POP divided by content of phytosterols at baseline before treatment ×100. To facilitate comparison between the studies, the reported units for the POP and total phytosterol contents were converted into mg/100 g and g/100 g of foods, respectively. Phytosterol contents in foods are expressed as free phytosterol equivalent (i.e., 1 g free phytosterols = 1.6 g phytosterol esters). For conciseness, only the mean values of the reported POP contents and the initial phytosterol contents in the foods from each individual study were extracted and are presented in this review.

### 2.4 Pooled analysis of POP in subgroups of foods with added free plant sterols, plant sterol, and plant stanol esters

To summarise the POP contents of foods with added free plant sterols (FPS), PSE and plant stanol esters (PAE) that were either non‐treated or underwent different storage, pan‐frying or oven‐heating procedures, we conducted a pooled analysis of POP contents in subgroups. For this, FPS‐, PSE‐, and PAE‐enriched foods were classified into four subgroups: 1) Non‐treated foods referring to POP contents of non‐treated foods and basal data of foods before any storage or heat treatment, 2) foods stored at different storage conditions, 3) foods pan‐fried at 160–200°C for 5–10 min, and 4) foods oven‐heated at 140–200°C for either 5–30 min or 60–120 min. Both mean and median values were calculated. The 90% quantile values for POP and ORP was calculated only for those subgroups with data points >15, as this allows proper statistical modelling. The 90% quantile was calculated using software JMP 12.1®.

## 3 Results

### 3.1 Overview of eligible publications

In total, 14 eligible publications reporting POP data of various foods with added phytosterols are included 15, 17, 21, 22, 23, 24, 25, 26, 27, 28, 29, 30, 31, 32; these are presented in Tables 1–3. As can be recognized, these studies differ considerably in the applied experimental conditions. Firstly, various types of foods, that is, butter, margarine, vegetable oils, milk, chocolate, fruit beverages, and milk beverages with added phytosterols were investigated. Secondly, these foods were enriched with different amounts of phytosterols ranging from 0.3 g/100 g in milk 30 to 14.7 g/100 g in margarine 15. Thirdly, the chemical form of phytosterols used for enrichment were either FPS, PSE, or PAE. Fourthly, the spectrum of the individual groups of POP, that is, 7‐OH‐, 7‐keto‐, epoxy‐ and triol derivatives of phytosterols measured differ between the studies. In some studies, all four POP groups were measured, while other studies only measured two or three groups. In addition, most of the studies used GC‐MS methods for measuring POP, while other studies used GC‐FID, GC‐MS/MS, HPLC‐ESI‐MS, or LC–GC‐MS for determining POP contents. In the following sections, we aim to assess the effects of FPS, PSE, and PAE added to foods next to factors related to storage and heat processing on the POP formation and contents in these foods.

### 3.2 POP formation and contents in foods with added FPS

Four studies investigated foods with added various amounts of FPS (0.5–8 g/100 g food) 21, 22, 23, 24. These FPS‐enriched foods were either non‐treated, stored at various temperatures, or processed under different heating conditions (Table 1).

**Table 1:** POP contents in foods with added free plant sterols (FPS) non‐treated, stored under different storage conditions or heated under different temperature and time conditions^a^

References	Analytical methods	POP measured^f^	Food format (matrix)	Experimental conditions			Results				
				Treatment	Temperature (°C)	Time	Before treatment			After treatment	
							PS (g/100 g)	POP (mg/100 g)	ORP (%)	POP (mg/100 g)	ORP^d^ (%)
Non‐treated foods											
Alemany‐Costa 2012 [21]	GC‐MS/MS	7‐OH 7‐Keto Epoxy	Fruit beverage	—	—	—	1.3	0.7	0.05	—	—
			Milk‐fruit beverage	—	—	—	1.4	0.8	0.06	—	—
Stored foods											
Gonzalez‐Larena 2015 [22]	GC‐MS	7‐OH 7‐Keto Epoxy Triol	Milk	Storage	4–37^c^	8–24 wk^c^	0.7	0.4	0.05	0.6	0.09
			Milk fruit beverage	Storage	4–37^c^	8–24 wk^c^	0.7	0.2	0.03	0.4	0.06
			Fruit beverage	Storage	4–37^c^	8–24 wk^c^	0.7	0.5	0.07	0.4	0.06
								
Soupas 2006 [23]	GC‐FID & GC‐MS	7‐OH 7‐Keto Epoxy Triol	Milk powder	Storage	RT^b^	12–36 wk^c^	7	1.4	0.02	1.8	0.03
					38	12–36 wk^c^				2.5	0.04
					4	6–24 wk^c^	0.5	0.2	0.04	0.19	0.04
					RT^b^				0.04	0.15	0.03
Foods heated at high temperature >100°C											
Soupas 2007^e^ Experiment 1 [24]	GC‐FID & GC‐MS	7‐OH 7‐Keto Epoxy Triol	Rapeseed oil	Pan‐frying	160	5 min	8.0	Not detectable^g^	<0.01	8.0	0.10
						10 min				32.0	0.40
					180	5 min				40.0	0.50
						10 min				144.0	1.80
					200	5 min				72.0	0.90
Soupas 2007^e^ Experiment 2 [24]	GC‐FID & GC‐MS	7‐OH 7‐Keto Epoxy Triol	Rapeseed oil	Pan‐frying	160	5 min	8.0	2.3	0.03	11.9	0.15
						10 min				23.9	0.30
			Liquid margarine			5 min		2.3	0.03	24.6	0.31
						10 min				73.1	0.91
			Butter			5 min		2.0	0.03	34.4	0.43
						10 min				121.4	1.52
			Rapeseed oil	Pan‐frying	180	5 min	8.0	2.3	0.03	35.5	0.44
						10 min				111.2	1.39
			Liquid margarine			5 min		2.3	0.03	74.4	0.93
						10 min				187.4	2.34
			Butter			5 min		2.0	0.03	118.3	1.48
						10 min				296.1	3.70

a Values are rounded to one decimal.

b RT, room temperature.

c Mean values for the indicated range of conditions are presented as there were no virtual differences in POP contents in foods.

d ORP (oxidation rates of PS) calculated as POP content in foods divided by baseline phytosterol content ×100.

e Different batches of rapeseed oil were used in the two experiments, hence POP contents before heat treatment varied.

f 7‐OH (7‐hydroxyphytosterols); Epoxy (5,6‐epoxyphytosterols); 7‐Keto (7‐ketophytosterols); Triol (phytosterol‐3,5,6 triol).

g Limit of determination of POP was 0.02 mg/100 g, which was used for the calculation of ORP.

#### 3.2.1 Effect of FPS content on POP formation in foods

POP contents of foods before any treatment that contained a small amount of added FPS (0.5–1.4 g/100 g) were compared with POP contents of food with a larger amount of added FPS (7–8 g/100 g). The POP contents of foods with added small vs. larger amounts of FPS were ≤0.8 mg/100 g and ≤2.3 mg/100 g, respectively, with a corresponding ORP of ≤0.07% for both groups.

#### 3.2.2 Effect of storage conditions on POP formation

Two studies investigated POP formation in fruit beverage, milk‐based fruit beverage and milk powder with varying added amounts of FPS (0.5–7 g/100 g food), which were stored at either room temperature or at 4–38°C for 6–36 wk. These foods contained ≤1.4 mg/100 g of POP, with ORP being ≤0.07% at baseline and ≤2.5 mg/100 g of POP with ORP being ≤0.09% at end of storage (Table 1).

#### 3.2.3 Effect of heating temperature on POP formation

When rapeseed oil was heated at 160, 180, or 200°C for 5 min (Experiment 1, Soupas et al. 24), the net increases in POP above baseline were 8.0, 40.0, and 72.0 mg/100 g oil, respectively. Accordingly, the ORP also increased from ≤0.01% (baseline) to 0.10, 0.50, and 0.90%, respectively, indicating that the heat‐induced increase in POP formation was associated with an increase in ORP. Heat treatment at 160 and 180°C for 10 min as compared to 5 min generated more POP with an increased ORP.

#### 3.2.4 Effect of food matrix on POP formation

As summarised in Table 1 the food matrix, for example, rapeseed oil, (liquid) margarine or butter, has a strong impact on POP formation. When these fats were pan‐fried at 180°C for 10 min, the highest POP content was found in butter (296.1 mg/100 g, ORP 3.70%) followed by liquid margarine (187.4 mg/100 g, ORP 2.34%) and rapeseed oil (111.2 mg/100 g, ORP 1.39%), all above the corresponding baseline POP contents of 2.0–2.3 mg/100 g.

### 3.3 POP formation and contents in foods with added PSE

Twelve studies investigated POP formation in different food formats including milk, milk powder, (milk) fruit beverage, chocolate, vegetable oils, (liquid) margarine and butter 15, 17, 21, 23, 24, 25, 26, 27, 28, 29, 30, 31. These foods were enriched with PSE (free PS equivalents ranging from 0.3 to 13.3 g/100 g of food) and treated under different storage or heating conditions or non‐treated (Table 2).

**Table 2:** POP contents in foods with added plant sterol sterols (PSE) non‐treated, stored under different storage conditions or heated under different temperature and time conditions^a^

References	Analytical method^c^	POP measured^g^	Food format (matrix)	Experimental conditions			Results				
				Treatment	Temperature (°C)	Time	Before treatment			After treatment	
							PS (g/100 g)	POP (mg/100 g)	ORP (%)	POP (mg/100 g)	ORP^d^ (%)
Non‐treated foods											
Alemany‐Costa 2012 [21]	GC‐MS/MS	7‐OH 7‐Keto Epoxy	Fruit beverage	—	—	—	1.4	0.8	0.06	—	—
			Milk‐fruit beverage	—	—	—	1.9	0.8	0.04	—	—
Baumgartner 2013 [15]	GC‐MS	7‐OH 7‐Keto	Margarine	—	—	—	13.3	3.37	0.03	—	—
Conchillo 2005 [25]	TLC & GC‐MS	7‐HO 7‐Keto Epoxy Triol	Spread	—	—	—	6	4.7	0.07	—	—
Husche 2011[17]	GC‐MS	7‐OH 7‐keto	Margarine	—	—	—	12.7	2.1	0.02	—	—
Grandgirard 2004 [26]	GLC‐MS	7‐OH 7‐Keto Epoxy Triol	Margarine	—	—	—	8.0	6.8	0.085	—	—
Johnsson 2006 [27]	GC‐FID & GC‐MS	7‐OH 7‐Keto Epoxy Triol	Margarine	—	—	—	Unknown	1.2	—	—	—
Stored foods											
Julien‐David 2014 [28]	HPLC‐ESI‐MS	7‐OH 7‐Keto Epoxy	Margarine	Storage	RT^b^ and dark	35 d	10.5	0.1	<0.01	0.4	<0.01
					RT^b^ and sunlight	35 d		0.1	<0.01	1.3	0.01
Soupas 2006 [23]	GC‐FID & GC‐MS	7‐OH 7‐Keto Epoxy Triol	Milk powder	Storage	4	6–24 wk^c^	0.5	0.2	0.04	0.2	0.04
					RT^b^	6–24 wk^c^		0.2	0.04	0.2	0.04
Botelho 2014 [29]	GC‐MS	7‐OH 7‐Keto Epoxy	Chocolate	Storage	30	20 wk	7.1	6.9	0.10	7.1	0.10
			Chocolate	Storage plus anti‐oxidants	30	20 wk	8.0	7.8	0.10	8.3	0.10
Foods heated at moderate temperature <100°C											
Menendez‐Carreno 2008 [30]	GC‐MS	7‐OH 7‐Keto Epoxy	Milk	Schaal oven	65	24 h	0.3	0.2	0.07	0.3	0.10
				Microwave oven	69	1.5 min				0.4	0.13
				Microwave oven	83	2 min				0.3	0.10
				Heating plate	87	15 min				0.3	0.10
Foods heated at high temperature >100°C											
Soupas 2007^e^ Experiment 1 [24]	GC‐FID & GC‐MS	7‐OH 7‐Keto Epoxy Triol	Rapeseed oil	Pan‐frying	160	5 min	8.0	8.0	0.10	16.0	0.20
						10 min				40.0	0.50
					180	5 min				40.0	0.50
						10 min				144.0	1.80
					200	5 min				72.0	0.90
Soupas 2007^e^ Experiment 2 [24]	GC‐FID & GC‐MS	7‐OH 7‐Keto Epoxy Triol	Rapeseed oil	Pan‐frying	160	5 min	8.0	3.9	0.05	9.3	0.12
						10 min				16.9	0.21
			Liquid margarine			5 min		4.0	0.05	13.3	0.17
						10 min				31.8	0.40
			Butter			5 min		3.4	0.04	15.3	0.19
						10 min				38.1	0.48
			Rapeseed oil	Pan‐frying	180	5 min	8.0	3.9	0.05	24.2	0.30
						10 min				58.5	0.73
			Liquid margarine			5 min		4.0	0.05	29.1	0.36
						10 min				66.9	0.84
			Butter			5 min		3.4	0.04	51.5	0.64
						10 min				123.1	1.54
Julien‐David 2014^f^ [28]	HPLC‐ESI‐MS	7‐OH 7‐Keto Epoxy	Margarine	Oven heating	140	5 min	10.5	0.2	<0.1	0.2	<0.01
						10 min				0.2	<0.01
						15 min				0.2	<0.01
						30 min				0.2	<0.01
						60 min				1.1	0.01
						120 min				412	3.92
			Margarine	Oven heating	170	5 min	10.5	0.2	<0.01	0.2	<0.01
						10 min				0.3	<0.01
						15 min				1.6	0.02
						30 min				18.9	0.18
						60 min				244.7	2.33
						120 min				503.6	4.80
			Margarine	Oven heating	200	5 min	10.5	0.2	<0.01	0.3	<0.01
						10 min				7.2	0.07
						15 min				58.9	0.56
						30 min				228.0	2.17
						60 min				384.3	3.66
						120 min				295.8	2.82
Scholz 2015 [31]	LC–GC‐MS	7‐OH 7‐Keto Epoxy	Margarine	Oven heating	180	30 min	7.5	37	0.49	42.0	0.56
						60 min				150.0	2.00
						90 min				484.0	6.45
						120 min				831.0	11.08

a Values are rounded to one decimal.

b RT, room temperature.

c Mean values for the indicated range of conditions are presented as there was no virtual differences in POP contents.

d ORP (oxidation rates of PS) were calculated as POP content in foods divided by baseline phytosterol contents ×100.

e Different batches of rapeseed oil were used in the two experiments, hence POP contents before heat treatment varied.

f Oxidized PSE were converted to POP equivalents using a converting factor of 0.6.

g 7‐OH (7‐hydroxyphytosterols); Epoxy (5,6‐epoxyphytosterols); 7‐Keto (7‐ketophytosterols); Triol (phytosterol‐3,5,6 triol).

#### 3.3.1 Effect of PSE content on POP formation

POP contents of foods before any treatment with a low amount of added PSE (0.3–1.9 g/100 g) were compared with the POP contents of foods with larger amounts of added PSE (6–13.3 g/100 g). The foods with added low amounts of PSE contained ≤0.8 mg/100 g of POP (mean 0.5 mg/100 g), while the foods with larger amounts of added PSE contained ≤37 mg/100 g POP (mean 6.8 mg/100 g), with a corresponding ORP of <0.10% for both groups. These data indicate that increasing PS contents was associated with an increase in POP contents, while the corresponding ORP did not increase accordingly.

#### 3.3.2 Effect of storage conditions on POP formation

In milk powder with added PSE (0.5 g/100 g) and stored at either 4°C or at room temperature for up to 24 wk, no detectable increase in POP content was found. When PSE‐enriched chocolate and margarine (7.1‐8 g/100 g) were stored at 30°C for 20 wk, POP contents increased by 0.2–0.5 mg/100 g but ORP at ≤0.10% was unchanged. Similarly, in PSE‐added margarine (10.5 g/100 g) and stored at room temperature with or without exposure to sunlight for 35 days, POP contents increased only by 0.3 mg/100 g and 1.2 mg/100 g, respectively, above baseline values 28. In all these stored food samples, ORP was ≤0.10%.

#### 3.3.3 Effect of heating temperature on POP formation

When milk with added PSE (0.3 g/100 g) was heated at 65–87°C for either short (1.5–15 min) or longer time (24 h), POP contents increased by ≤0.2 mg/100 g compared to baseline values and ORP was maintained at about 0.13% 30. Heating temperatures in the range of 140–200°C had a strong impact on POP formation in foods with added PSE (Table 2). Moreover, an interaction between heating temperature and heating time within the range of 5–120 min was apparent. For example, as reported by Soupas et al. (Experiment 2) 24, POP contents significantly increased by 5.3–119.7 mg/100 g above the basal POP contents (about 4 mg/100 g) in PSE‐enriched rapeseed oil, liquid margarine and butter that were pan‐fried at 160–180°C for 5–10 min. ORP increased from <0.1% up to 1.5% after heating 24. Similarly, Julien‐David et al. 28 showed that in margarine with added PSE (10.5 g/100 g) and heated in an oven at temperatures of 140–200°C for 5–120 min, POP contents were non‐linearly increased. At the first 15 min of heating at 140–200°C, the maximal net increase of POP was 58.9 mg/100 g, corresponding to an ORP of 0.56%. Heating margarine at 140, 170, or 200°C for 30–120 min, POP formation increased more rapidly (Table 2). The highest POP content in margarine heated at 180°C for 120 min in an oven was reported by Scholz et al. 31 with 831 mg/100 g, and a corresponding ORP of 11.08%

#### 3.3.4 Effect of food matrix on POP formation

The food matrix, for example, rapeseed oil, liquid margarine or butter, again had a strong impact on POP formation (Table 2). For example, pan‐frying at 180°C for 10 min, resulted in the highest amount of POP in butter (123.1 mg/100 g, ORP 1.54%) followed by liquid margarine (66.9 mg/100 g, ORP 0.84%) and rapeseed oil (58.5 mg/100 g, ORP 0.73%).

### 3.4 POP formation and contents in foods with added PAE

Four studies investigated the effect of either non‐treatment, various storage conditions or pan‐frying on the formation and contents of POP in various foods including butter, rapeseed oil, (liquid) margarine and milk powder with added 0.5–14.7 g/100 g plant stanols in the form of PAE 15, 23, 24, 32 (Table 3).

**Table 3:** POP contents in foods with added plant stanol esters (PAE) non‐treated, stored under different storage conditions or heated under different temperature and time conditions^a^

References	Analytical methods	POP measured^e^	Food format (matrix)	Experimental conditions			Results				
				Treatment (tools)	Temperature (°C)	Time	Before treatment			After treatment	
							PS (g/100 g)	POP (mg/100 g)	ORP (%)	POP (mg/100 g)	ORP^c^ (%)
Non‐treated foods											
Baumgartner 2013 [15]	GC‐MS	7‐OH 7‐Keto	Margarine	—	—	—	14.7	0.54	<0.01	—	—
Stored foods											
Soupas 2006 [23]	GC‐FID & GC‐MS	6‐OH 7‐OH 15‐OH	Milk powder	Storage	4	6–24 wk^b^	0.5	0.02	<0.01	0.03	0.01
					RT					0.03	0.01
Rudzinska 2014 [32]	GC‐FID and GC‐MS	7‐OH 7‐Keto Epoxy Triol	Margarine	Stored on open Petri dish	4	6–18 wk^b^	7.9^f^	25.5^f^	0.32	38.0	0.48
					20	6–18 wk^b^				67.1	0.85
Foods heated at high temperature >100°C											
Soupas2007^d^ Experiment 1 [24]	GC‐FID & GC‐MS	6‐OH 7‐OH 15‐OH	Rapeseed oil	Pan‐frying	160	5 min	8.0	8.0	0.10	8.0	0.10
						10 min				8.0	0.10
					180	5 min				8.0	0.10
						10 min				8.0	0.10
					200	5 min				8.0	0.10
Soupas 2007^d^ Experiment 2 [24]	GC‐FID & GC‐MS	6‐OH 7‐OH 15‐OH	Rapeseed oil	Pan‐frying	160	5 min	8.0	4.3	0.05	4.5	0.06
						10 min				4.5	0.06
			Liquid margarine			5 min		3.6	0.05	4.8	0.06
						10 min				5.5	0.07
			Butter			5 min		0.4	0.01	0.7	0.01
						10 min				1.2	0.02
			Rapeseed oil	Pan‐frying	180	5 min	8.0	4.3	0.05	4.7	0.06
						10 min				6.1	0.08
			Liquid margarine			5 min		3.6	0.05	4.9	0.06
						10 min				6.3	0.08
			Butter			5 min		0.4	0.01	2.0	0.03
						10 min				3.9	0.05

a Values are rounded to one decimal. RT, room temperature.

b Mean values for the indicated range of conditions are presented as there was no virtual differences in POP contents.

c ORP (oxidation rate of PS) was calculated as POP content in foods divided by baseline phytosterol contents ×100.

d Different batches of rapeseed oil were used in the two experiments, hence POP contents before heat treatment varied.

e 6‐OH‐ (6‐hydroxyphytostanols) 7‐OH, (7‐hydroxyphytostanols), 15‐OH‐ (15‐hydroxyphytostanols).

f Ratio of plant stanols to plant sterols = 70:8 and POP values are the sum of oxidised derivatives of both plant sterols and stanols.

#### 3.4.1 Effect of PAE content on POP formation in foods

Before any treatment, in milk powder with a low (0.5%) PAE content, POP content was low (0.02 mg/100 g, ORP <0.01%), while in foods with higher PAE contents (7.9–14.7%) higher amounts of POP were found (0.4–25.5 mg/100 g, with ORP ranging from <0.01% to 0.32%). It has to be noted that in the study of Rudzinska et al. 32, total POP is reported which did not distinguish between oxidation products of plant stanols from those of plant sterols leading to an overestimation of the actual amount of plant stanol oxidation products. In the other studies 15, 24, foods with 8.0–14.7 g/100 g of added PAE contained 0.4–0.8 mg/100 g of POP with the corresponding ORP of ≤0.10%.

#### 3.4.2 Effect of storage conditions on POP formation

Soupas et al. 23 reported that in milk powder with added PAE (0.5 g/100 g) and stored at 4°C or at room temperature for 6–24 wk only trace amounts of (0.01 mg/100 g) of POP were found and ORP was 0.01%. In contrast, Rudzinska et al. 32 found that POP contents in margarine with added PAE (7.9 g/100 g) were significantly increased from 25.5 mg/100 g (ORP 0.3%) to 38.0 mg/100 g (ORP 0.48%) when stored at 4°C for 6 wk and to 67.1 mg/100 g (ORP 0.85%) when stored at 20°C for 6 wk, respectively. These data indicate that storage conditions increased POP contents in PAE‐enriched margarine, and more POP were formed at 20°C as compared to 4°C storage. However, this study also showed that prolonged storage from 6 up to 18 wk at either 4 or 20°C did not further increase POP formation (Table 3). As described above, the study by Rudzinska et al. 32 overestimated the content of plant stanol oxidation products because only total POP are reported.

#### 3.4.3 Effect of heating temperature on POP formation

In rapeseed oil, liquid margarine and butter pan‐fried at 160–180°C for 5–10 min, POP contents were only moderately increased, with a net increase of 0.2–3.5 mg/100 g and a corresponding ORP ≤0.08% (Experimental 2) 24. Similar results (Experimental 1) were found when rapeseed oil was pan‐fried at 160–200°C for 5–10 min, with a net increase in POP contents of <0.1 mg/100 g and ORP maintained at 0.10%. These data indicate that PAE are more resistant than FPS or PSE to POP formation under pan‐frying conditions.

### 3.5 Pooled analysis of POP formation and contents in subgroups of foods with added FPS, PSE, and PSA

The pooled data for the four defined subgroups of foods described in the section 2.4 are summarised in Table 4. The data reported by Rudzinska et al. 32 are not included in this analysis due to the mix of oxidation products of plant stanols and of plant sterols. Mean values differ from the median values regarding POP contents of non‐treated, stored and heated foods, indicating that in these foods the reported POP data are skewed. Hence, the median is considered a better parameter to summarize the reported POP contents. The median POP contents of non‐treated foods were 0.7, 3.4, and 3.6 mg/100 g for FPS‐, PSE‐, and PAE‐enriched foods with a median ORP of 0.03, 0.06, and 0.05%, respectively. The 90% quantile of POP of non‐treated PSE‐ enriched foods is 13.8 mg/100 g, with a corresponding ORP of 0.38%. In foods stored at temperatures of 4–38°C for 5–24 wk, median POP contents were 0.4, 0.9, and 0.03 mg/100 g for FPS, PSE, and PAE‐enriched foods with ORP of 0.04, 0.04, and 0.06%, respectively. In foods pan‐fried at 160–200°C for 5–10 min, median POP contents were 72.0, 38.1, and 4.9 mg/100 g for FPS, PSE, and PAE‐enriched foods, with corresponding ORP of 0.9, 0.48, and 0.06%, respectively. The 90% quantile of POP were 209.1, 127.3, and 8.0 for FPS‐, PSE‐, and PAE‐enriched foods with a corresponding ORP of 2.61, 1.59, and 0.10%, respectively. In PSE‐enriched foods oven‐heated at 140–200°C for 5–30 min, POP contents were very low, with a median of 0.3 mg/100 g, and corresponding ORP of <0.01%, while with oven‐heating at 140–200°C for 60–120 min, POP contents were relatively high with a median POP content of 384.3 mg/100 g, and a corresponding ORP of 3.66%.

**Table 4:** Pooled analyses of POP contents and ORP in subgroups of foods with added phytosterols before food storage and heat treatment, and end of storage, pan‐frying or oven heating

			POP contents (mg/100 g)			ORP (%)		
Form of phytosterols added	Food formats/storage or heating conditions	Data points (n)	Mean	Median	90% quantile^a^	Mean	Median	90% quantile^a^
Non‐treated and baseline data (before food storage or heat treatment)								
FPS	Milk powder, milk, beverage, chocolate, rapeseed oil, liquid margarine, and butter	11	1.0	0.7	—	0.04	0.03	—
PSE	Milk, beverage, milk powder, chocolate, rapeseed oil, liquid margarine, and butter	17	5.3	3.4	13.8	0.10	0.06	0.38
PAE	Milk powder, chocolate, rapeseed oil, liquid margarine, and butter	7	6.1	3.6		0.08	0.05	—
End of storage								
FPS	Milk, beverage, milk powder stored at 4–38°C for 6–24wk	7	0.9	0.4	—	0.05	0.04	—
PSE	Milk powder, margarine and chocolate stored at 4–30°C for 5–24 wk	6	2.9	0.9	—	0.05	0.04	—
PAE	Milk powder stored at 4°C‐RT^b^ for 6–24 wk	2	0.03	0.03	—	0.06	0.06	—
Pan‐frying								
FPS	Rapeseed oil, liquid margarine and butter pan‐fried at 160–200°C for 5–10 min	17	82.8	72.0	209.1	1.04	0.90	2.61
PSE	Rapeseed oil, liquid margarine and butter pan‐fried at 160–200°C for 5–10 min	17	46.5	38.1	127.3	0.58	0.48	1.59
PAE	Rapeseed oil, liquid margarine and butter pan‐fried at 160–200°C for 5–10 min	17	5.2	4.9	8.0	0.07	0.06	0.10
Oven‐heating								
PSE	Margarine heated at 140–200°C for 5–30 min	13	27.6	0.3	—	0.27	<0.01	—
PSE	Margarine heated at 140–200°C for 60–120 min	9	367.4	384.3	—	4.12	3.66	—

FPS, free plant sterols; PSE, plant sterol esters; PAE, plant stanol esters.

a 90% quantile values were only calculated with data points >15.

b RT, room temperature.

## 4 Discussion

### 4.1 Summary of data on POP formation in foods with added phytosterols

This review systematically evaluates the available data regarding POP contents of a spectrum of typically consumed foods with added phytosterols, such as milk products, fruit drinks, chocolate, butter, (liquid) margarine and vegetable oils and addresses factors that influence POP formation. Some of these foods were heat‐treated resembling typical household cooking procedures. The reported POP contents showed large variation which can be explained by the presence or absence of several factors that impact POP formation. These factors and their combinations that play a role in POP formation and subsequently POP contents of foods are: 1) the level of PS‐enrichment, 2) heating temperature and time, 3) the chemical form of phytosterols, that is, FPS, PSE, and PAE being added to the foods, and 4) the food format/matrix itself.

The absolute POP contents of foods were positively associated with their phytosterols contents (Tables 1–3). Phytosterols are precursors of POP. Increasing the initial phytosterol contents of foods increased food POP contents. However, increasing the amount of phytosterols does not increase relative POP formation as indicated by a low ORP (median ≤0.06%) in foods with non‐treatment or before any treatment (baseline POP data), which was independent of the amounts of phytosterols added to the foods. Therefore, phytosterol‐enrichment per se has only a modest effect on the percentage of phytosterol oxidation in non‐treated foods.

Typical storage temperatures (4–38°C) have only negligible effects on POP formation in foods with added FPS and PSE, as indicated by low median POP contents (<1 mg/100 g) and ORP (≤0.06%) at the end of food storage (Table 4).

Heating temperature, heating time and their interaction are important determinants of POP formation in foods. The net increase in the POP contents in the studied foods was positively associated with heating temperature and duration of heating although a large heterogeneity exists among the reported studies. This is, at least partly, due to the different heating conditions applied in the individual studies. Pan‐frying at temperatures of 160–200°C for 5–10 min represents typical household cooking conditions; therefore, POP formation under these conditions is considered to reflect POP contents of typical household heat‐treated foods (Table 4). Further, FPS, PSE or PAE were shown to be differently resistant to thermal oxidation, with the order of resistance being PAE > PSE > FPS and with a corresponding ORP of 0.06, 0.48, and 0.90%, respectively (Table 4). An implication of these findings is that PAE and PSE are a preferred form for food enrichment other than FPS regarding a lesser formation of POP.

Heating margarine in an oven for up to 30 min hardly affected POP formation, but heating for 60–120 min significantly increased POP formation up to (median) 384.3 mg/100 g, with a corresponding ORP of 3.66% (Table 4). Of note, in these studies 28, 31 only margarine alone was heated for a long time not being mixed with dough, hence not reflecting typical and realistic household baking. Mixing margarine with other ingredients into a dough may reduce the formation of POP by, for example, reducing exposure to air oxygen. Therefore, these study results may overestimate the POP contents in real baked foods. Further studies are required to confirm or reject such speculation. Fat‐based products like margarine with added phytosterols may also be used for basting meats during roasting. In such a scenario, the formed POP may partly be distributed between the food and the residual fat, which is either not consumed or partly consumed when used for making a gravy. Therefore, the real intake of POP from frying or roasting foods using phytosterol enriched fat‐based products need further study.

The impact of the food format or matrix on POP formation is demonstrated by comparing the different POP contents in rapeseed oil, liquid margarine and butter, which were all studied under the same experimental conditions (Tables 1–3). POP formation was highest in heated butter followed by heated liquid margarine and lowest in heated rapeseed oil. These results are in agreement with the finding that cholesterol in heated butter was highly sensitive to oxidation, as apparent by a cholesterol oxidation rate of 12.5%, as reviewed by Savage et al. 35. The reasons behind such an impact of food matrix on POP formation under heat treatment are not well understood. Further studies are required to explore whether the amount of saturated, mono‐ and polyunsaturated fatty acids, next to the presence of components with pro‐ or antioxidative activity may affect POP formation.

### 4.2 Estimation of daily POP intake resulting from consumption of foods with added phytosterols

Daily POP intake can be estimated based on the daily phytosterol intake from foods with added phytosterols multiplied by the ORP in these foods.

Regarding daily phytosterol intake of individuals who use foods with added phytosterols for lowering their elevated plasma cholesterol concentrations three different scenarios were considered. Firstly, actual daily phytosterol intake data from the most recent European post launch monitoring survey were used. 36. A previous survey published in 2006 reported mean daily phytosterol intakes per one‐member households in European countries ranging from 0.24 g/day to 0.96 g/day 37. Intake data reported in 2013 are similar with mean daily phytosterol intakes per one‐member households ranging from 0.28 g in France to 0.75 g in the UK 36. Secondly, a daily phytosterol intake of 2 g/d for lowering LDL‐cholesterol as part of a healthy diet as for example, recommended by the International Atherosclerosis Society 38 was used. And thirdly we used the advised upper daily phytosterol intake of 3 g/d that should not be exceeded according to the European regulatory requirement 39.

For the ORP of foods with added phytosterols we used data summarized in this review for estimating POP intake. As aforementioned, ORP of non‐heated and stored foods and heat‐treated, that is, pan‐fried foods differ greatly. To calculate the daily POP intake we considered a worst‐case scenario by using the largest ORP values, that is, the 90% quantile values of 0.38% for non‐heated or stored foods with added PSE and the 90% quantile values of 2.61, 1.59, and 0.10% for pan‐fried foods with added FPS, PSE or PAE, respectively.

Based on these phytosterol intake and ORP data, the estimated daily POP intake is summarized in Table 5. For non‐heated and stored foods with added PSE, the estimated daily POP intakes are in the range of 2.9–11.4 mg/d according to the different scenarios of daily phytosterol intakes (PS intakes from 0.75 g/d to 3 g/d). For pan‐fried foods, the estimated daily POP intakes from foods with added PAE, PSE, and FPS are 3.0, 47.7, and 78.3 mg/d, respectively for the highest phytosterol intake of 3 g/d. Considering that the actual phytosterol intake by consumers using foods with added phytosterols based on post launch monitoring data was 0.75 g/d 36, the estimated POP intakes from heated foods with added PAE, PSE and FPS are 0.8, 11.9, and 20.0 mg/d, respectively.

**Table 5:** Estimated daily POP intakes in mg/day based on different intake scenarios

			Phytosterol intake from foods with added phytosterols according to different intake scenarios		
Forms of phytosterols added	ORP%		Post‐launch monitoring survey of intake of physterol‐enriched foods [36]^a^ (0.75 g/d)	IAS recommendation for phytosterol intake for a cholesterol lowering benefit [38] (2 g/d)	EFSA advised upper dose of phytosterol intake [39] (3 g/d)
Non‐heated foods					
PSE	90% quantile	0.38	2.9	7.6	11.4
Pan‐fried foods					
FPS	90% quantile	2.61	20.0	52.2	78.3
PSE	90% quantile	1.59	11.9	31.8	47.7
PAE	90% quantile	0.10	0.8	2.0	3.0

FPS, free plant sterols; PSE, plant sterol esters; PAE, plant stanol esters.

a Mean value of daily phytosterol intake from phytosterol‐enriched foods per one‐person household in the UK, which was the highest value in European countries.

PSE and PAE are the most common formats of phytosterols being added to foods currently available in the market. Therefore, the estimated daily POP intakes being below 47.7 mg/d (0.8–3.0 mg/day for PAE and 11.9–47.7 mg/d for PSE based on the different phytosterol intake scenarios) may reflect more the real situation of POP intakes of consumers who would use foods with added phytosterols for household cooking. It should be noted that the actual daily phytosterol intake in European countries based on post launch monitoring surveys is in fact much lower being below 1 g/d than the recommended 2 g/d and advised upper 3 g/d phytosterol intake, hence a POP intake of 47.7 mg/d clearly reflects a worst‐case scenario estimation.

Recently, Scholz et al. 9 proposed to use ORP values of 0.1% and 1% to estimate daily POP intake from non‐heated and heated foods, respectively. They estimated that POP intakes were 3 mg/d and 30 mg/d for non‐heated and heated foods bases on 3 g added phytosterols. Evidently, in the present review we selected a more conservative approach by using an ORP of 0.38% and 1.59% based on the 90% quantile value for the calculation. Thus, the estimated POP intakes in this review are higher than that estimated by Scholz et al. 9.

### 4.3 Estimated POP intake in humans in comparison to toxicological data from animal studies

PSE is the most common form of phytosterols being added to foods and most study data available addressed POP contents of foods enriched with PSE, compared to foods with added FPS or PAE. Therefore, we further compare the estimated POP intake from foods with added PSE with data from animal studies.

The estimated upper daily POP intakes from non‐heated and pan‐fried foods enriched with PSE are in a range of 11.9–47.7 mg/d, equivalent to 0.16–0.69 mg/kg BW/d (for an adult of 70 kg body weight). A 90‐day feeding study in rats established the “No Observed Effect Level” (NOEL) as being 128 mg/kg BW/d for males and 144 mg/kg BW/d for females 40. Taking the lowest NOEL value into account, a daily intake of up to 47.7 mg/d of POP seems unlikely to result in adverse effect in humans because it is about 186‐times lower than the NOEL obtained in rats. If considering FPS as a source for enrichment of foods, the estimated upper daily POP intake is 78.3 mg, which is about 115‐times lower than the NOEL.

The estimated upper daily POP intake from foods enriched with PSE and FPS is 0.69 and 1.1 mg/kg BW/d, respectively. Such POP intake are much lower than for instance the dose of 30 mg/kg BW/d of POP used in the rate study by Plat et al. 12, in which the POP‐containing diet was shown to be atherogenic as compared to the control diet. In contrast, other animal studies with mice and hamsters did not show such a pro‐atherogenic effect with POP doses of 25–74 mg/kg BW/d 14, 19, 20. Further studies addressing the potential pro‐atherogenic effects of POPs in animals and, if feasible, in humans with realistic daily human POP intakes would be helpful.

### 4.4 Limitations of the reported data and further study needs

At current, the number of studies reporting data on POP formation and contents in foods with added phytosterols is small, covering also only a relatively narrow spectrum of foods and with a large diversity of reported data regarding phytosterols and POP contents in these foods. In addition, the performed heat treatments were mostly under experimental laboratory conditions. Whether the so generated POP contents indeed reflect realistic POP contents in foods prepared under typical household cooking and baking conditions needs to be confirmed. Finally, but also importantly it cannot be excluded that differences in the analytical methods used to measure POP in the reported studies may contribute to the large variation in the reported POP contents in foods. Therefore, an attempt to harmonize the methods for POP analysis in foods is encouraged.

This review summarizes POP contents in foods with added phytosterols. POP contents in customary consumed foods without added phytosterols have not yet been systematically evaluated. POP formation derived from the oxidation of naturally existing phytosterols in foods such as vegetable oils could serve as a background value for the dietary intake of POP with a habitual diet. As previously reviewed by Otaegui‐Arrazola et al. 7, various amounts of POP in the range of 0.01–10.2 mg/100 g are found in vegetable oils. Using traditional vegetable oils for cooking and baking may also increase POP contents in foods, which has not yet been systematically evaluated. Intestinal absorption of POP in humans is not known, but can be considered low based on absorption rates (<15.9%) of POP observed in rats 14, 18. Hence, it is reasonable to assume that daily POP intake of less than 78.3 mg POP might not have a major impact on plasma POP concentrations. However, further studies addressing plasma concentrations, after intake of realistic amounts of POP, are required.

## 5 Conclusions

Several influencing factors, such as the amount and chemical form of phytosterols added to foods, but especially the heating temperature and time, impact POP formation and contents in foods. Non‐heated and stored foods with added phytosterols contained minor amounts of POP (median ≤3.6 mg/100 g) with a corresponding ORP of <0.10%. Under common household cooking conditions, that is, pan‐frying and oven heating at temperatures of 140–200°C for 5–30 min, foods with added PSE (the most common form of phytosterols being added to foods) contained (median) ≤38.1 mg/100 g POP with a corresponding ORP of ≤0.48%. In the considered worst‐case scenario (assuming a 3 g/d phytosterol intake and a 90% quantile ORF value of 1.59%), the daily upper POP intake was estimated to be 47.7 mg/d with PSE, equivalent to 0.69 mg/kg BW/d for an adult with 70 kg body weight. Considering FPS the daily upper POP intake would be 78.3 mg, equivalent to 1.12 mg/kg BW/d. Such a POP intake would be 115‐times lower than the established NOEL (128 mg/kg BW/d) observed in rats. Taken together, this review contributes to a better understanding of POP formation and contents in non‐heated and heat‐treated foods with added phytosterols in free‐ or esterified forms and the health implication of their intake.

6

The authors gratefully acknowledge Dr. Rouyanne Ras for her assistance in the systematic literature search and Dr. Ursula Garczarek for her contribution in performing the statistical assessment of the data.

*The authors have no conflict of interest. All authors are employed by Unilever R&D Vlaardingen. Unilever markets food products with added plant sterols*.
